# Improved measurement of tinnitus severity: Study of the dimensionality and reliability of the Tinnitus Handicap Inventory

**DOI:** 10.1371/journal.pone.0237778

**Published:** 2020-08-25

**Authors:** Elżbieta Gos, Adam Sagan, Piotr H. Skarzynski, Henryk Skarzynski

**Affiliations:** 1 Department of Teleaudiology and Screening, World Hearing Center, Institute of Physiology and Pathology of Hearing, Warsaw, Poland; 2 Department of Market Analysis and Marketing Research, Faculty of Management, Cracow University of Economics, Cracow, Poland; 3 Heart Failure and Cardiac Rehabilitation Department, Faculty of Medicine, Medical University of Warsaw, Warsaw, Poland; 4 Institute of Sensory Organs, Kajetany, Poland; 5 Department of Otorhinolaryngosurgery, World Hearing Center, Institute of Physiology and Pathology of Hearing, Warsaw, Poland; University of Copenhagen, DENMARK

## Abstract

**Objective:**

The Tinnitus Handicap Inventory (THI) is widely used in clinical practice and research as a three-dimensional measure of tinnitus severity. Despite extensive use, its factor structure remains unclear. Furthermore, THI can be considered a reliable measure only if Cronbach’s alpha coefficient and Classical Test Theory is used. The more modern and robust Item Response Theory (IRT) has so far not been used to psychometrically evaluate THI. In theory, IRT allows a more precise evaluation of THI’s factor structure, reliability, and the quality of individual items.

**Method:**

There were 1115 patients with tinnitus (556 women and 559 men), aged 19–84 years (M = 51.55; SD = 13.28).

The dimensionality of THI was evaluated using several models of Confirmatory Factor Analysis and an Item Response Theory approach. Exploratory non-parametric Mokken scaling was applied to determine a unidimensional and robust scale. Several IRT polytomous models were used to assess the overall quality of THI.

**Results:**

The bifactor model had the best fit (RMSEA = 0.055; CFI = 0.976; SRMR = 0.040) and revealed one strong general factor and several weak specific factors. Mokken scaling generated a reliable unidimensional scale (Loevinger’s *H* = 0.463). In order to refine THI we propose that five items be removed. The IRT Generalized Partial Credit Model generated good parameters in terms of item location (difficulty), discrimination, and information content of items.

**Conclusion:**

Our findings support the use of THI to evaluate tinnitus severity in terms of it being a reliable unidimensional scale. However, clinicians and researchers should rely only on its overall score, which reflects global tinnitus severity. To improve its psychometric quality, several refinements of THI are proposed.

## Introduction

In recent years there has been increasing interest among clinicians and healthcare providers in assessing patients’ health status using Patient Reported Outcome Measures (PROMs). A PROM instrument is any report of the status of a patient’s health that originates directly from the patient [1]. PROMs have been defined as health questionnaires which evaluate aspects of a patient’s health from the patient’s perspective [2].

PROMs are useful in clinical practice for diagnosis, choice of treatment, and monitoring changes. There is evidence that the use of PROMs improves patients’ satisfaction, allows monitoring of response to treatment, and detects unrecognized problems [3]. In clinical trials they serve as primary or secondary endpoints [4], and they are used in health systems and in health policymaking for assessing and improving quality of care [5, 6]. The scope of PROMs’ application is still expanding [7], and efforts have been made recently to ensure that the methodology of PROM use is clinically meaningful, valid, and reliable [8–11]; only then can they serve as effective instruments in enhancing healthcare quality.

PROMs are particularly useful in assessing subjective disorders which are not apparent to others but which are registered only through the complaints of the sufferers–and tinnitus is one such disorder. Tinnitus is the subjective perception of sound without any external acoustic stimulation, and is perceived as ringing in the ears, hissing, chirping, buzzing, or other sounds [12–14]. Its prevalence ranges from 4% to 15% in adults [15], and 6% to 34% in children [16–18]. Tinnitus is accompanied by a broad range of negative emotional symptoms, and significantly impacts on quality of life [19, 20]. Because of the limited effectiveness of audiological assessment and psychoacoustic measurement, self-reported rating scales and questionnaires are widely used in evaluating the severity of individually perceived tinnitus [21–23], where severity is defined as the level of distress or impact that tinnitus has on the person [24]. There is no other option for measuring tinnitus severity other than with self-reporting measures (primarily multi-item questionnaires), which need to have acceptable psychometric quality.

There are many questionnaires used for assessing tinnitus severity [23]. The Tinnitus Handicap Inventory (THI) stands out among them–it is the most commonly used tool which has been validated in the largest number of languages [25]. THI was created to evaluate the impact of tinnitus on daily living [26], and is used as a screening tool for psychiatric disorders [27], and as an outcome measure for evaluating treatment effects in clinical trials [21, 28–30]. There is a brief, time-efficient screening version which consists of only 10 items, and this has greatly increased the use of THI [31].

Although THI is a widespread tool, its factor structure remains unclear. Newman and colleagues originally postulated three factors–the Emotional, Functional, and Catastrophic subscales–but they were based on item content, not on factor analysis [26]. Factor analysis for THI was first reported for a Danish version of THI, but the study sample comprised only 50 tinnitus patients [32]. Exploratory factor analysis did not confirm a three-factor solution, indicating that only the THI total score should be used as a valid measure of tinnitus severity (not the scores on the three subscales). In 2003, Baguley and Andersson conducted exploratory factor analysis of THI in a group of 196 patients, and the analysis gave strong support for a unifactorial structure [33]. To date, more than a dozen factor structure validation studies of THI have been published, with study groups ranging from 50 [32, 34] to 373 patients [35]. The majority of these studies failed to demonstrate a three-factor structure [36–38], although two of them did support the original three-factor solution [35, 39]. In particular, the German study seems very strong: in its confirmatory factor analysis it used a large sample of 373 tinnitus patients [35]. The findings showed that a three-factor model gave a better fit than a unidimensional model, and indicated that the three subscales of THI (Functional, Emotional, Catastrophic) were each valid and provided three distinct dimensions of tinnitus severity.

It is worth noting that work so far has used only a Classical Test Theory (CTT) approach, whereas a more modern and robust approach is now available–Item Response Theory (IRT). In this context, the factor structure of THI is not just an academic exercise but an important problem in clinical practice. It is crucial for a clinician or researcher to know which factor structure (three- or unidimensional) is appropriate to the situation and be confident they can rely on each subscale score or only on the total THI score.

The second issue which is critical to psychometric quality is reliability. The most popular index of reliability is Cronbach’s alpha coefficient, which is based on CTT [40]. All studies concerning psychometric properties of THI report alpha for both total scale and for subscales. Reliability across studies appears to be very high, mostly above 0.90. Across almost all studies, alpha for the Functional and Emotional subscales ranged from 0.8 to 0.9, while for the Catastrophic subscale it was lower, about 0.6–0.7. However, Cronbach’s alpha coefficient has numerous limitations [41–43], and other more robust model-based indices of reliability have recently been proposed. Reliability estimates within CTT has some limitations–they are dependent on the particular sample and measurement error is the same across all level of the ability. IRT overcomes these limitations treating reliability as precision of measurement independent of the particular sample and enabling estimation of measurement error at any given level of a latent trait.

The present study has three goals:

1. To examine the theoretical structure of tinnitus severity as measured by THI. Our starting hypothesis is that a unidimensional model best accounts for the structure of a measured construct.
2. To determine the reliability of THI in a model-based approach which has so far not been used in psychometric studies of THI.
3. To give guidance for a potential refinement of THI using Item Response Theory.

## Method

### Design

Our retrospective study used data from patients admitted to a tertiary referral ENT center in Poland over the period July 2015 to September 2018. Patients had reported problems with tinnitus as a primary complaint or secondary to hearing loss, and filling in THI was part of the standard diagnostic evaluation. Records of patients were retrospectively screened to check compliance with the eligibility criteria: age above 18 years, duration of tinnitus at least 1 month, documented hearing thresholds based on clinical pure tone audiometry, and a completed Tinnitus Handicap Inventory. The Institutional Review Board approved the protocol of the study (approval no. KB IFPS 18/2018). Due the retrospective nature of our evaluation, no written consent from the participants were gathered.

### Measures

The Tinnitus Handicap Inventory (THI) comprises 25 items grouped into three subscales: Functional, Emotional, and Catastrophic. The Functional subscale (11 items) deals with limitations caused by tinnitus in the areas of mental, social, and physical functioning. The Emotional subscale (9 items) concerns affective responses to tinnitus, e.g. anger, frustration, depression, anxiety. The Catastrophic subscale (5 items) probes the most severe reactions to tinnitus, such as loss of control, inability to escape from tinnitus, and fear of having a terrible disease. For each item a patient can respond with a “yes” (scored 4 points), “sometimes” (2 points), or “no” (0 points). The responses are summed within each subscale and for the total scale. The higher the score, the greater the perceived tinnitus severity [26]. The Polish version of THI validated by Skarzynski et al. [38] was used in this study.

### Participants

There were 1115 individuals (556 women and 559 men); their mean age was 51.6 years (SD = 13.3) and ranged from 19 to 84 years. The period of suffering from tinnitus varied from 1 month to 50 years (M = 6.6; SD = 7.7). Most frequently, the tinnitus was bilateral (57%), while 26% of the patients reported tinnitus in the left ear and 17% in the right.

### Data analysis

The first step was to evaluate the dimensionality of THI, and here four CFA models were used: a unidimensional CFA, a second-order CFA, a bifactor CFA, and a three-dimensional CFA model with correlated factors. Weighted Least Square estimation with means and variance adjustment of Chi-square statistics (WLSMV) and Theta and Delta parameterization were applied. Taking into account that the THI items are ordinal categorical variables, polychoric correlation coefficients were used. The overall fit of a CFA model was considered adequate if its Root Mean Square Error of Approximation (RMSEA) was < 0.05, the Comparative Fit Index (CFI) was > 0.95, and the Standardised Root Mean Square Residual (SRMR) < 0.05 [44].

Model-based reliability was assessed by McDonald’s omega and the *H*-index, and the average variance extracted [45]. McDonald’s omega was calculated as both omega total (ω) and omega hierarchical (ω_H_), and for the bifactor model omega hierarchical of the subscales (ω_HS_) and Percentage of Reliable Variance (PRV) were also calculated [46]. An omega value above 0.80 was considered high [47]. Omega hierarchical above 0.75, in conjunction with a PRV above 75%, indicates a scale’s unidimensionality. Omega hierarchical subscale reflects the reliability of a subscale after controlling for the variance due to the general factor [48]. Average Variance Extracted (AVE) refers to the variance explained by a construct due only to measurement error. Fornel and Larcker stated it should be at least 0.5 [49]. The *H*-index is a measure of maximal reliability for an optimally-weighted scale, i.e. when each item contributes different information to the global score [50, 51]. The *H*-value was expected to have a minimum of 0.7.

Additional measures of dimensionality were applied in the bifactor model. Explained Common Variance (ECV) is an indicator of unidimensionality, with high ECV indicating a strong general factor compared to group factors [52]. Item Explained Common Variance (IECV) shows item-level variation attributed to a general factor [53]. ECV was used in conjunction with Percent of Uncontaminated Correlations (PUC). ECV > 0.70 and PUC > 0.70 suggest that a given construct is unidimensional [47]. Average Relative Parameter Bias (ARPB) occurs when multidimensionality is ignored and a unidimensional model is specified [47]. An ARPB less than 10–15% is considered acceptable [54].

The second step involved exploring non-parametric Mokken scaling to check for the monotonicity of items. Selection of the best items for unidimensional parametric IRT modeling was carried out via an automated item selection procedure using a genetic algorithm. In terms of the IRT approach, the scalability of the THI scale was measured using Loevinger’s *H* [55]. If the item scalability coefficients *H*_ij_ > 0, *H*_i_ > 0.3, and *H* > 0.3 then this suggests a reliable, cumulative scale.

In the third step, three IRT polytomous models were used to assess unidimensional THI scale quality: the Rasch Model for polytomous items, the Generalized Partial Credit Model (GPCM, an extension of the Rasch model) with parameters for item discrimination and adjacent-category response functions [56], and the Graded Response Model (for ordered polytomous categories of a Likert scale and with cumulative category response functions) [57]. The overall fit was checked using the M2 statistic [58]. Marginal reliability was computed, given an estimated model and a prior density function; marginal reliability above 0.7 suggests an acceptable scale. The local independence assumption was checked using Yen’s *Q*_3_ statistic based on correlation of the residuals for a pair of items [59]. The final scale was developed on the basis of model-based reliability, item goodness of fit, and item information functions.

The sample size was calculated using power 0.80 and alpha level 0.05, assuming 3 latent variables, 25 observed variables, and an anticipated effect size of 0.1. The required minimum sample was 823 individuals. Statistical analyses were performed with IBM SPSS Statistics v.24, Mplus 8.2, and the mirt, ltm, eRm, and mokken libraries of the R package.

## Results

### Basic statistics

Descriptive statistics for the THI items and its subscales are summarized in Table 1. The majority of correlations between individual items and the total score were above 0.5, making the whole scale seem reliable.

**Table 1:** Descriptive statistics for THI.

	Yes (%)	Sometimes (%)	No (%)	M	SD	Corrected item–total correlation	Cronbach’s alpha if item deleted
THI 1 F	33.3	43.5	23.2	2.20	1.49	0.68	0.939
**THI 2** F	33.5	30.8	35.7	1.96	1.66	0.37	0.943
THI 3 E	29.8	39.0	31.2	1.97	1.56	0.65	0.939
THI 4 F	18.0	31.7	50.3	1.35	1.52	0.63	0.940
THI 5 C	17.2	30.6	52.2	1.30	1.51	0.69	0.939
THI 6 E	42.7	42.2	15.1	2.55	1.42	0.59	0.940
THI 7 F	35.2	33.1	31.7	2.07	1.64	0.49	0.941
**THI 8** C	66.5	20.3	13.2	3.07	1.43	0.48	0.941
THI 9 F	27.3	27.7	45.0	1.64	1.66	0.66	0.939
THI 10 E	26.0	38.2	34.9	1.84	1.57	0.69	0.939
THI 11 C	24.0	33.5	42.5	1.63	1.59	0.46	0.942
THI 12 F	30.5	34.9	34.6	1.92	1.61	0.75	0.938
**THI 13** F	22.5	34.0	43.5	1.58	1.57	0.67	0.939
THI 14 E	30.4	43.6	26.0	2.09	1.50	0.73	0.938
THI 15 F	28.3	32.5	39.2	1.78	1.63	0.57	0.940
THI 16 E	35.2	36.5	28.3	2.14	1.59	0.64	0.939
THI 17 E	17.1	25.2	57.7	1.19	1.53	0.61	0.940
THI 18 F	17.7	42.0	40.3	1.55	1.46	0.69	0.939
**THI 19** C	69.3	19.7	11.0	3.17	1.36	0.42	0.942
THI 20 F	35.9	39.4	24.7	2.22	1.54	0.69	0.939
THI 21 E	31.2	37.4	31.4	2.00	1.58	0.75	0.938
THI 22 E	20.8	25.9	53.3	1.35	1.60	0.63	0.940
THI 23 C	22.2	37.6	40.2	1.64	1.54	0.72	0.938
**THI 24** F	45.5	22.0	32.5	2.26	1.75	0.37	0.943
THI 25 E	29.2	27.3	43.5	1.71	1.68	0.67	0.939
	Range			M	SD	Cronbach’s alpha	Cronbach’s alpha if items 2,8,13,19,24 were deleted
Functional	0–44			20.53	11.71	0.875	0.865
Emotional	0–36			16.84	10.30	0.893	0.893
Catastrophic	0–20			10.81	5.17	0.731	0.696
THI total	0–100			48.18	25.27	0.942	0.942

Capital letters represent items contained on the subscales: F–Functional, E–Emotional, C- Catastrophic.

Corrected item-total correlation is a correlation between the item and the scale score that excludes this item.

Items excluded in subsequent analysis are in bold.

### Dimensionality of CTT- and IRT-based measurement models

Before testing multidimensional models, CFA unidimensional analyses of the Functional, Emotional and Catastrophic subscales were conducted using WLSMV method.

For Functional subscale: χ^2^ (44) = 295.14; p < 0.001; RMSEA (Root Mean Square Error Of Approximation) = 0.072; CFI (Comparative Fit Index) = 0.978; SRMR (Standardized Root Mean Square Residual) = 0.042. After controlling for correlated errors (based on modification index) items THI7 with THI20, and THI7 with THI2, χ^2^ (42) = 247.06; p < 0.001; RMSEA = 0.066; CFI = 0.982; SRMR = 0.038.

For Emotional subscale: χ^2^ (27) = 234.24; p < 0.001; RMSEA = 0.083; CFI = 0.983; SRMR = 0.035.

After controlling for correlated errors items THI3 with THI14, THI25 with THI17 and THI25 with THI22, χ^2^ (24) = 111.02; p < 0.001; RMSEA = 0.057; CFI = 0.993; SRMR = 0.023.

For Catastrophic subscale: χ^2^ (5) = 82.81; p < 0.001; RMSEA = 0.118; CFI = 0.967; SRMR = 0.045.

After controlling for correlated errors items THI8 with THI19, the fit drastically has been improved: χ^2^ (4) = 5.83; p < 0.001; RMSEA = 0.020; CFI = 0.999; SRMR = 0.012.

**Fig 1:**
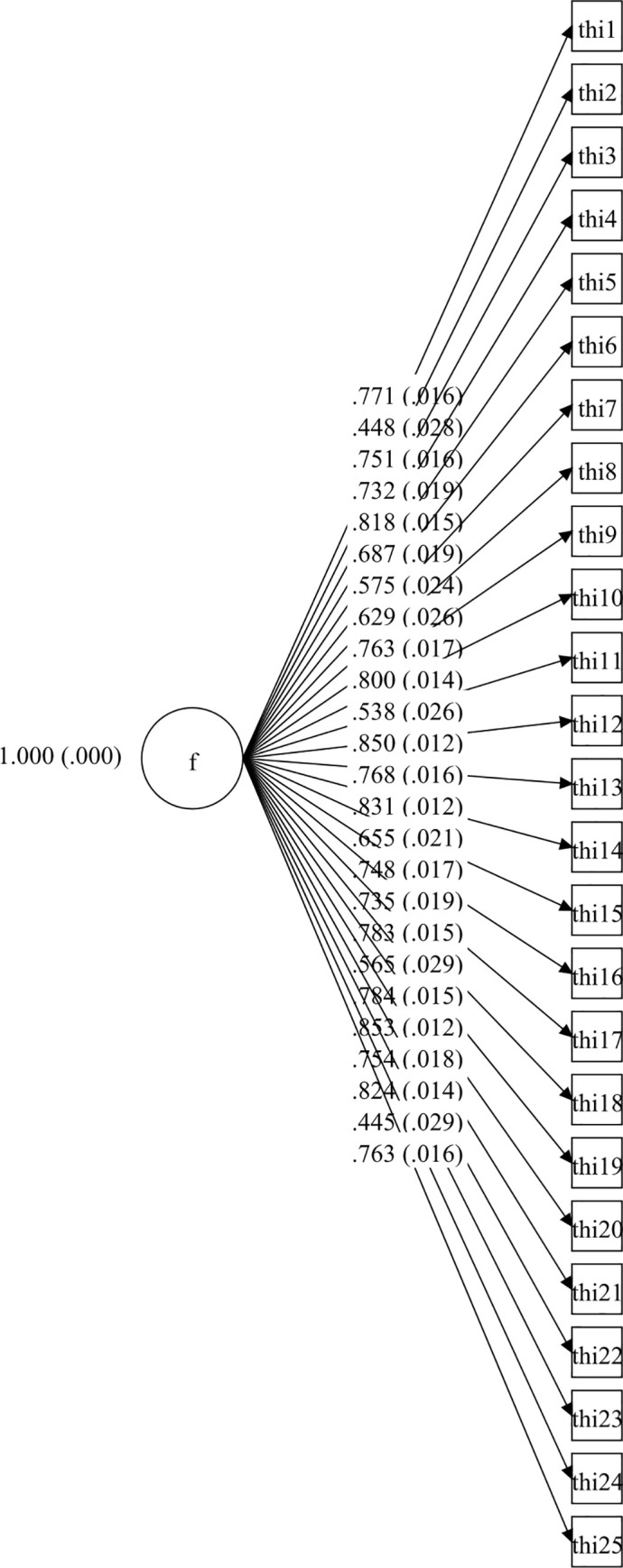
Unidimensional CFA model of Tinnitus Handicap Inventory.

**Fig 2:**
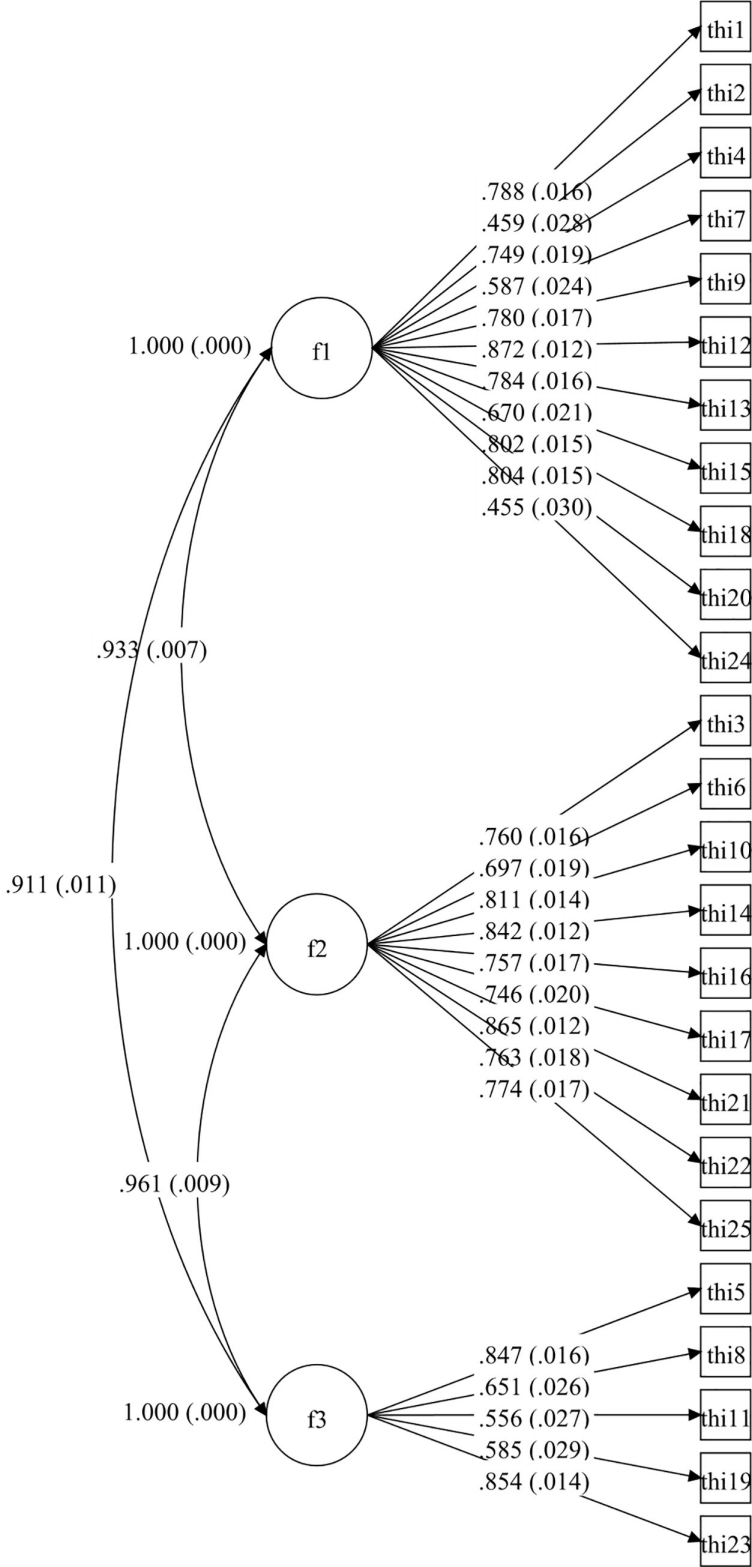
Three-dimensional CFA model of Tinnitus Handicap Inventory.

**Fig 3:**
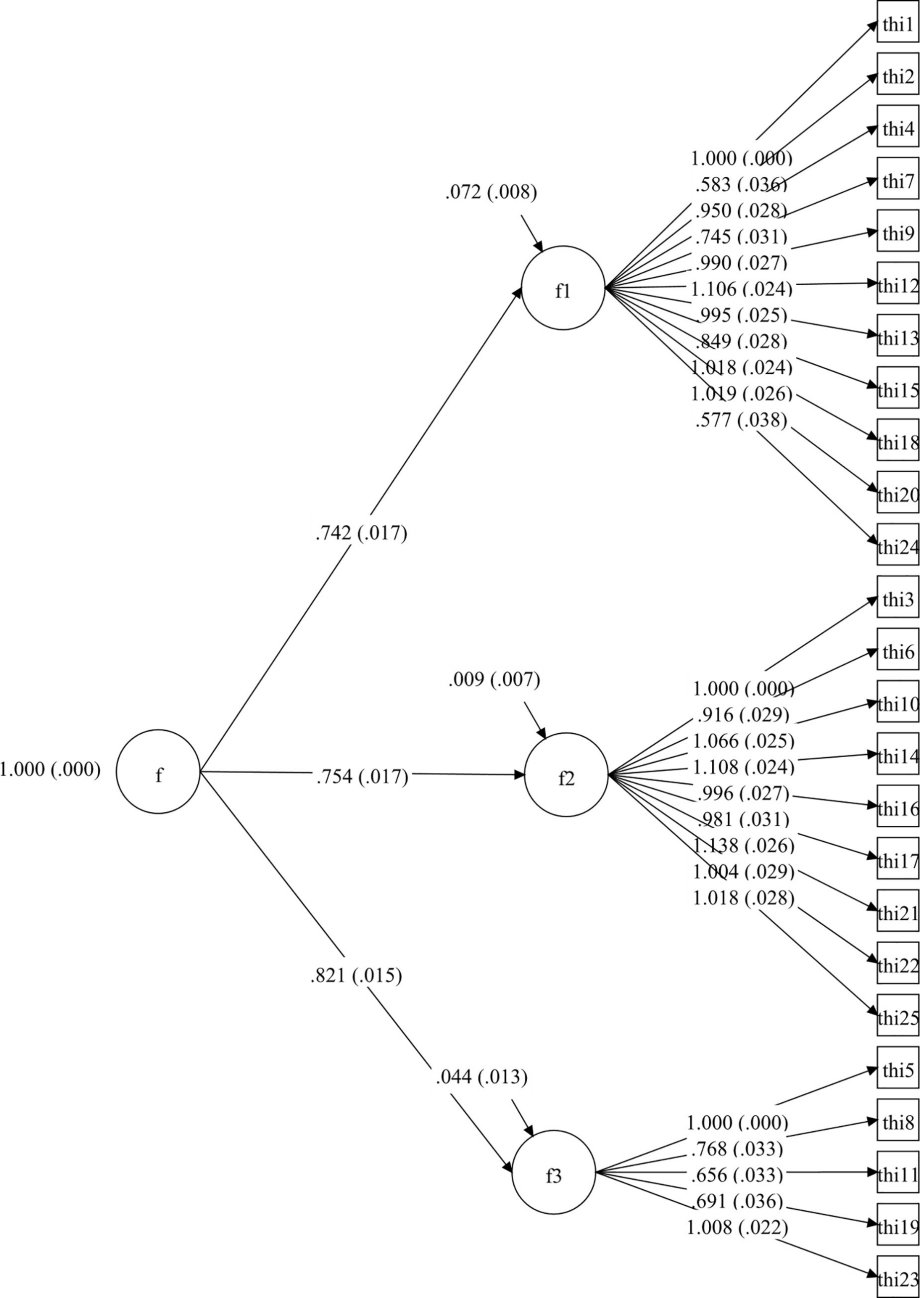
Second-order CFA model of Tinnitus Handicap Inventory.

**Fig 4:**
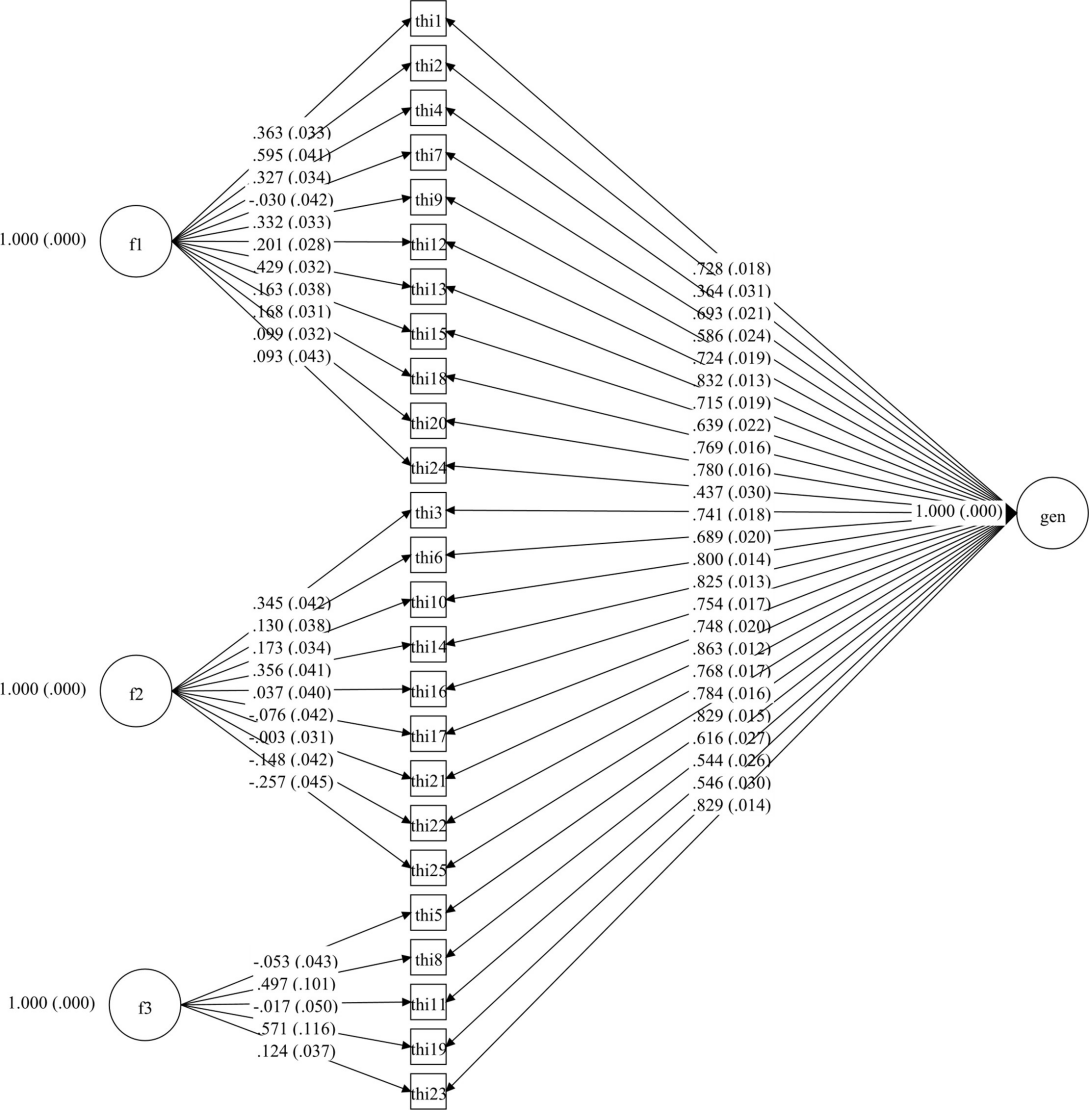
Bifactor CFA model of Tinnitus Handicap Inventory.

Afterwards, four CFA models for the whole THI were tested and they are set out in Figs 1–4.

Results of dimensionality analysis and comparison of models of goodness of fit are shown in Table 2.

**Table 2:** Goodness of fit for various THI models.

**CTT factor models**					
**Model**	**Chi-square**	**Df**	**RMSEA**	**CFI**	**SRMR**
Unidimensional	1701.362	275	0.068	0.960	0.051
Correlated factors	1522.900	272	0.064	0.965	0.048
Second-order	1493.146	272	0.064	0.964	0.049
Bifactor	1101.318	250	0.055	0.976	0.040
**Model comparison**	**Delta Chi-square**	**Delta df**	**Delta RMSEA**	**Delta CFI**	**Delta SRMR**
Bifactor vs Correlated factors	391.752 (p<0.001)	22	0.009	0.011	0.008
Correlated factors vs Unidimensional	148.837 (p<0.001)	3	0.013	0.016	0.003
**IRT models**					
**Model**	**M2**	**df**	**RMSEA**	**CFI**	**SRMR**
Unidimensional					
GPCM	1637.937	248	0.071	0.967	0.046
Rasch	1923.624	273	0.074	0.961	0.111
Correlated factors					
GPCM	-	-	-	-	-
Rasch	1750.035	266	0.071	0.965	0.113
Second-order					
GPCM	3496.855	245	0.109	0.9223	0.327
Rasch	-	-	-	-	-
Bifactor					
GPCM	822.086	193	0.054	0.984	0.164
Rasch	3075.998	265	0.094	0.933	0.409
**Model comparisons**	**Delta Chi**^**2**^	**Delta df**	**Delta RMSEA**	**Delta CFI**	**Delta SRMR**
Bifactor vs correlated factors Rasch (ANOVA)	-1527.237 (p>0.99)	1	0.023	0.032	0.296
Correlated factors vs unidimensional Rasch (ANOVA)	99.684 (p<0.001)	7	0.003	0.004	0.002
Bifactor vs unidimensional Rasch (ANOVA)	-1427.554 (p>0.99)	8	0.020	0.028	0.298

- Model failed to converge

RMSEA, Root Mean Square Error of Approximation; CFI, Comparative Fit Index; SRMR, Standardised Root Mean Square Residual

All the CTT models had acceptable goodness of fit, taking into account the values of fit indices. However, the bifactor model had a significantly better fit in comparison with the correlated factor model, which was slightly superior to the unidimensional model. In the family of IRT models, bifactor GPCM and unidimensional GPCM had the best fit (M2 statistic); however the SRMR of bifactor GPCM appeared to be too high. In summary, both CTT and IRT confirmatory models suggest a more detailed elaboration of the unidimensional and bifactor models is needed in order to verify the unidimensionality of THI.

### Model reliability

Reliability was evaluated for the two best models: unidimensional and bifactor. Results are gathered together in Table 3.

**Table 3:** Reliability of unidimensional and bifactor models.

	Uni-dimensional model	Bifactor model			
		F	E	C	THI total
ω	0.967	0.930	0.941	0.856	0.971
ω_H_		0.114	0.005	0.079	0.945
ω_HS_		0.021	0.001	0.003	-
H	0.970	0.570	0.300	0.460	0.950
AVE	0.550	0.187	0.166	0.199	0.581
PRV		0.123	0.006	0.093	0.973
ECV		0.066	0.029	0.033	0.872
IECV		THI1 = 0.801, THI2 = 0.272, THI3 = 0.822, THI4 = 0.818, THI5 = 0.996, THI6 = 0.966, THI7 = 0.997, THI8 = 0.605, THI9 = 0.826, THI10 = 0.955, THI11 = 0.999, THI12 = 0.945 THI13 = 0.735, THI14 = 0.843, THI15 = 0.939, THI16 = 0.998, THI17 = 0.990, THI18 = 0.955, THI19 = 0.478, THI20 = 0.984, THI21 = 1.000, THI22 = 0.964, THI23 = 0.978, THI24 = 0.956, THI25 = 0.903			
ARPB		0.03			
PUC		0.660			

F, Functional subscale; E, Emotional subscale; C, Catastrophic subscale; THI total, THI total score; ω, McDonald’s omega; ω_H_, omega hierarchical; ω_HS_, omega hierarchical subscale; H, Bentler’s index; AVE, Average Variance Extracted; PRV, Percentage of Reliable Variance; ECV, Explained Common Variance; IECV, Individual Explained Common Variance; ARPB, Average Relative Parameter Bias; PUC, Percent of Uncontaminated Correlations.

The unidimensional model had acceptable reliability. The bifactor model showed high overall and sub-dimension reliability; however a unidimensional solution was most strongly supported. Omega_H_ = 0.945 showed that total score predominantly reflects a single general factor. Omegas for the subscales scores seemed to demonstrate high reliability for the THI sub-factors, but low values of ω_HS_ indicated that almost all sub-scale score variance is due to the general factor and almost no variance is due to specific factors. It also indicated the heavy confounding of sub-scale reliability (reliabilities of sub-scales were overwhelmingly inflated). Also PRV values confirmed that the three subdimensions of the THI scale are questionable and suggest that the scale is undimensional. General ECV values also suggested the scale is unidimensional, with ECVs for sub-scales meaningless. The Difference ARP bias between the unidimensional scale and the general factor in the bifactor model was acceptable. Only PUC = 0.66 showed that there might be some multi-dimensionality in THI; however, it was not severe enough to disqualify the interpretation of the instrument as being primarily unidimensional. The individual explained common variance (IECV) indicated that almost all items well represent the unidimensional THI scale except items THI2 and THI19, which were less than 0.50. The best items for unidimensional THI scale having the highest IECV were THI21, THI11, THI16, THI7, THI5, THI6, THI17, THI20, THI23, THI22, and THI24.

In general, all criteria of dimensionality analysis (ω_H_, ω_HS_, PRV, ECV, PUC, and ARPB) gave sufficient support for scale unidimensionality. In the subsequent analysis, unidimensional IRT-based models are adopted to assess the monotonicity and quality of each THI item.

### Exploratory Mokken model of the unidimensional THI scale

**Fig 5:**
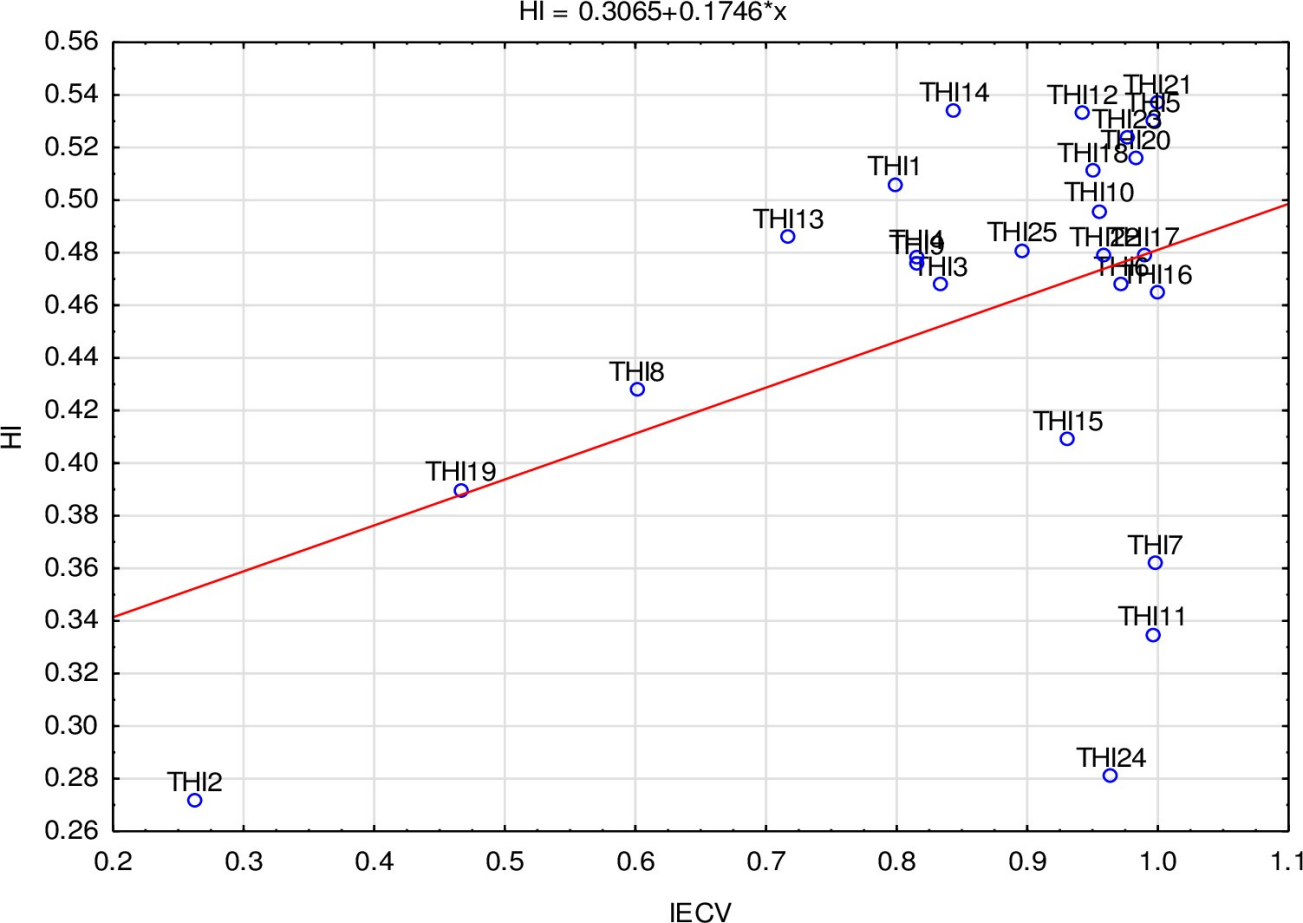
Relationship between *H*_i_ and IECV of THI items.

Having verified unidimensionality and the cumulative character of the THI scale, an exploratory nonparametric Mokken model was used to evaluate the scale’s monotonicity and to select items. All the item scalability coefficients *H*_ij_ between pairs of items were positive (*H*_ij_ > 0) and ranged between 0.127 (THI2–THI7) and 0.733 (THI5–THI10). THI2 and THI24 were regarded as the weakest items (*H*_i_ < 0.3). The Loevinger *H* for the total scale was 0.463 (SE = 0.011). Additional reliability measures (MS and LCRC) showed reliable unidimensional scale: MS = 0.909, LCRC = 0.949. Also, the Automated Item Selection Procedure (AISP) for the Mokken scale using a genetic algorithm confirmed unidimensionality, (except items THI2 and THI24). The relationships between *H*_i_ and IECV measures are plotted in Fig 5.

On the basis of existing sub-scales, model fit, *H*_i_, and IECV values we propose a shortened unidimensional THI scale that consists of only the “best” items. The selection is based on linear ordering (Hellwig method) and the geometric average of *H*_i_ and IECV scores. Items THI2, THI8, THI13, THI19, and THI24 were thus removed from the original scale, and the 20 remaining items were selected for unidimensional parametric polytomous IRT models.

### Item quality of IRT-based models

IRT analysis results for the three IRT models are summarized in Table 4.

**Table 4:** Goodness of fit of IRT-based models.

**Model level**	**Rasch model**	**GPCM**	**GRM**
M2; df (p-level)	1047.694; 169 (<0.001)	926.420;150 (<0.001)	901.572;150 (<0.001)
RMSEA	0.068	0.068	0.067
AIC	37705.55	37197.73	37125.99
Test information function value	63.71	63.71	65.69
Model marginal reliability	0.879	0.929	0.932
**Item level**	**Chi-square (p-level)**	**Chi-square (p-level)**	**Chi-square (p-level)**
THI1	46.295 (0.792)	43.238 (0.772)	43.054 (0.857)
THI3	41.764 (0.906)	43.262 (0.852)	42.900 (0.901)
THI4	49.804 (0.637)	49.285 (0.725)	51.550 (0.800)
THI5	75.261* (0.024)	62.993 (0.086)	55.121 (0.254)
THI6	72.026* (0.022)	69.026 (0.057)	64.774 (0.172)
THI7	164.566* (<0.001)	59.596 (0.598)	61.102 (0.614)
THI9	65.717 (0.227)	59.063 (0.436)	60.253 (0.467)
THI10	59.197 (0.360)	54.009 (0.360)	57.335 (0.284)
THI11	219.252* (0.000)	52.031 (0.911)	52.481 (0.918)
THI12	83.330* (0.016)	43.736 (0.686)	59.375 (0.197)
THI14	97.678* (<0.001)	75.718* (0.007)	78.870* (0.003)
THI15	116.421* (<0.001)	64.191 (0.400)	69.604 (0.325)
THI16	47.831 (0.773)	47.204 (0.763)	49.085 (0.732)
THI17	38.489 (0.933)	50.166 (0.727)	44.273 (0.923)
THI18	77.759* (0.019)	68.544* (0.042)	69.173* (0.046)
THI20	54.012 (0.512)	44.856 (0.679)	50.947 (0.593)
THI21	90.779* (0.002)	44.792 (0.523)	45.004 (0.556)
THI22	55.634 (0.413)	55.135 (0.470)	57.788 (0.557)
THI23	80.512* (0.027)	45.652 (0.610)	47.467 (0.574)
THI25	61.698 (0.312)	60.652 (0.380)	61.531 (0.421)

* significant at p<0.05

**Fig 6:**
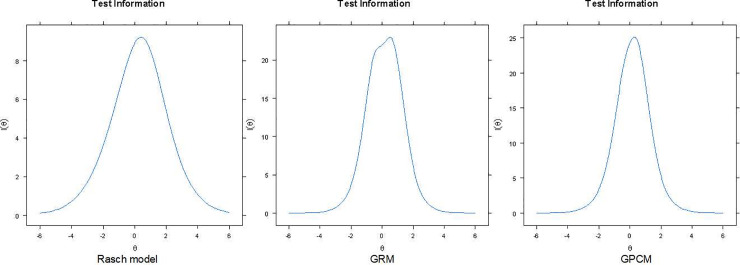
Test information curves of IRT models of THI (20 items).

The test information curves of compared models are given in Fig 6.

The Rasch model was rejected and the GPCM and GRM models seemed to be the most appropriate. The GPCM model was chosen for further analysis.

The reliability of all the models was above the threshold of 0.7 and between –2.5 and +2.5 standard deviations from the average level of the standardized latent trait. The GPCM model included 93.05% of respondents who fitted the model and it was selected for more detailed analysis of items and individual person’s reliability.

The Yen’s *Q*_3_ statistic was used to test the assumption of local independence. The mean value was –0.025 and *Q*_3_ ranged between –0.107 and 0.160. The mean *Q*_3_ value was less than the threshold value of 0.1 and indicated that the local independence assumption was valid. Additionally, correlations between standardized residuals correlations were calculated and they are gathered in Table 5. The mean value for residual correlations was -0.007 and they ranged between -0.5 to 0.14, and for only one pair of items it was rather high (-0.5).

**Table 5:** Correlations between standardized residuals (GPCM Model).

	THI1	THI3	THI4	THI5	THI6	THI7	THI9	THI10	THI11	THI12	THI14	THI15	THI16	THI17	THI18	THI20	THI21	THI22	THI23
THI1																			
THI3	-0.029																		
THI4	0.109	0.044																	
THI5	-0.065	0.089	0.120																
THI6	-0.104	0.082	-0.100	0.063															
THI7	0.075	0.075	-0.054	-0.054	0.086														
THI9	0.060	-0.047	0.077	-0.060	-0.085	-0.080													
THI10	-0.050	0.081	-0.100	0.135	-0.099	-0.028	0.087												
THI11	-0.039	-0.025	0.056	0.088	0.066	-0.015	-0.036	-0.054											
THI12	0.041	-0.076	-0.075	0.079	-0.105	-0.074	0.128	-0.121	-0.025										
THI14	-0.04	0.143	-0.029	-0.109	0.141	0.050	-0.069	0.133	-0.058	-0.097									
THI15	0.083	-0.043	0.072	0.067	-0.032	0.105	-0.079	-0.070	-0.039	-0.021	-0.041								
THI16	-0.095	0.034	-0.064	-0.079	0.124	-0.054	-0.067	-0.068	0.139	-0.031	-0.074	-0.055							
THI17	0.101	-0.053	0.071	-0.096	-0.089	-0.084	0.122	-0.082	-0.044	0.064	0.109	0.062	-0.026						
THI18	0.089	-0.049	-0.063	0.076	0.113	0.064	-0.040	-0.071	0.060	-0.075	-0.096	0.101	-0.060	0.069					
THI20	0.074	-0.087	-0.052	-0.060	-0.108	0.060	0.060	0.055	-0.033	-0.073	0.098	0.046	-0.072	-0.109	-0.072				
THI21	-0.082	-0.061	-0.071	-0.083	0.110	-0.066	-0.045	-0.129	0.050	0.080	-0.075	-0.062	0.116	0.041	-0.088	0.039			
THI22	-0.042	-0.065	0.060	0.109	-0.068	0.072	-0.078	-0.064	0.089	-0.089	-0.50	-0.082	0.078	-0.056	-0.081	-0.062	0.069		
THI23	-0.073	-0.082	-0.093	0.069	0.053	0.059	-0.081	-0.081	0.060	-0.094	-0.054	0.061	0.084	-0.062	0.079	0.085	0.086	0.091	
THI25	0.075	-0.053	0.114	-0.124	-0.042	-0.059	0.072	-0.094	0.069	0.056	-0.052	-0.054	-0.047	0.071	-0.061	-0.056	-0.059	0.088	-0.054

The parameters of the GPCM model are given in Table 6. Item locations (difficulties) were calculated as an average of threshold parameters for item response categories (for three item categories, two thresholds exist).

**Table 6:** Item parameters of GPCM model for THI (20 items).

	Model parameters				
Items	Threshold 1	Threshold 2	Item location	Discrimination	Info
THI1	-0.947	0.516	-0.216	1.671	3.34
THI3	-0.577	0.601	0.018	1.570	3.14
THI4	0.211	1.108	0.659	1.321	2.64
THI5	0.165	1.092	0.628	1.948	3.90
THI6	-1.482	0.169	-0.656	1.307	2.61
THI7	-0.408	0.219	-0.090	0.826	1.65
THI9	0.114	0.545	0.329	1.324	2.65
THI10	-0.437	0.715	0.139	1.852	3.71
THI11	0.144	0.869	0.506	0.703	1.40
THI12	-0.430	0.554	0.062	2.194	4.39
THI14	-0.782	0.600	-0.091	2.285	4.57
THI15	-0.102	0.565	0.231	1.006	2.01
THI16	-0.668	0.379	-0.144	1.479	2.96
THI17	0.559	1.037	0.798	1.334	2.67
THI18	-0.280	1.165	0.442	1.865	3.73
THI20	-0.843	0.392	-0.225	1.770	3.54
THI21	-0.556	0.543	-0.006	2.440	4.88
THI22	0.372	0.837	0.604	1.438	2.88
THI23	-0.265	0.894	0.314	2.128	4.26
THI25	0.051	0.459	0.255	1.394	2.79

**Fig 7:**
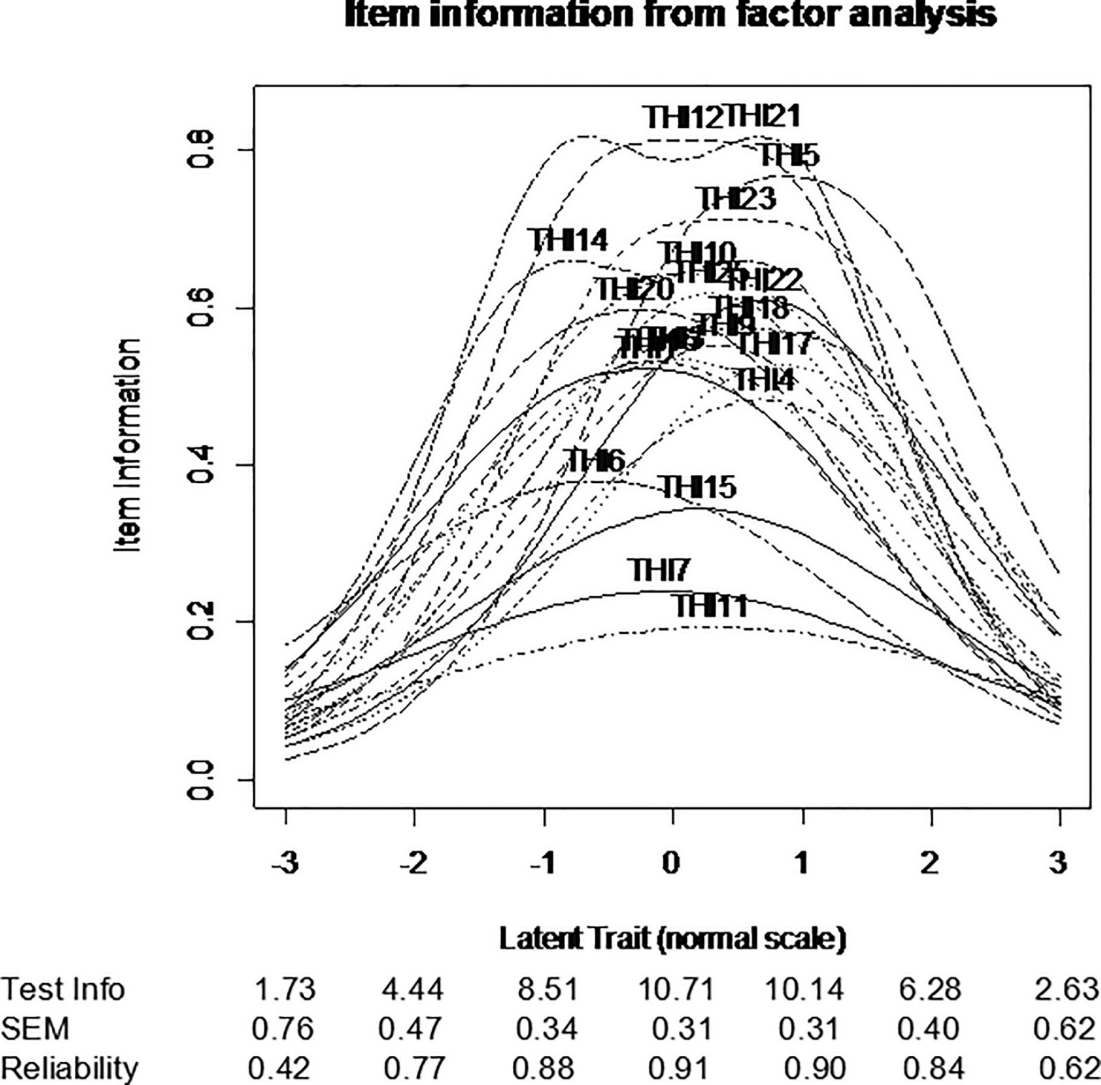
Item information functions.

Item difficulties ranged between –0.656 (THI6) and 0.798 (THI17), item discrimination between 0.703 (THI11) to 2.440 (THI21), and item information between 1.40 (THI11) and 4.88 (THI21). For those item information values between –2 and 2 standardized values of Θ (latent trait continuum), where the THI scale has the highest precision, the item information values were between 0.940 (THI11) and 4.74 (THI 21), which are shown in Fig 7.

## Discussion

Despite widespread use of THI, there are still doubts about its psychometric quality. The first doubt has to do with its unclear factor structure, which means it is not certain whether THI correctly gauges aspects of tinnitus severity. Originally, it was postulated that THI measures three domains of tinnitus severity: functional, emotional, and catastrophic. They were intended to be distinct, although strongly correlated [26].

Our findings do not support these assumptions. Our findings show that, for the clinical population, the original three-factor structure is not the best measure of tinnitus severity. Omega hierarchical sub-scale indices showed that the proportion of the total variance accounted for by the three subscales was, after controlling for the influence of general tinnitus severity, very small. Other indices (AVE, ECV, PUC, PRV, ARPB) showed that the common variance can be regarded as unidimensional, thus supporting one general factor and a unidimensional solution. These results are in line with our previous research [38] and they are also consistent with those obtained by others [32, 33, 36, 37]. This contrasts with the earlier German study of 373 tinnitus patients [35], which confirmed the three-factor structure of THI.

However, it should be noted that the German study compared only a general factor model and a first-order three-factor model. They did not consider a second-order three-factor model or a bifactor model. It is known that a bifactor model is useful for evaluating the validity of multi-item questionnaires which measure both the overall construct and its specific dimensions [47]. In our case, however, the results of bifactor modelling clearly demonstrated that there was a one factor solution. Our results demonstrate that THI should be considered a unidimensional scale, and that the Functional, Emotional, and Catastrophic subscales do not represent separate substantive latent traits. Instead, we believe these subscale share a large portion of overall general negative affectivity associated with tinnitus.

THI is generally considered to be a reliable tool. The claim about high reliability of THI subscales and overall score, demonstrated by several validating studies, is founded on the use of Cronbach’s alpha coefficient. But it is worth emphasizing, that reliability depends on a particular study population, while IRT offers in its place test information function, which shows the degree of precision at different values of the latent trait. Fig 7 clearly shows that the standard error of measurement (SEM) is the smallest in the middle of the scale and increases with higher and lower scores. So, the precision of measurement is the highest for the subjects with moderate tinnitus severity. When Cronbach’s alpha is embedded in CTT theory, it is assumed that SEM is constant along the scale, and this is, as we can see, an unfounded assumption. Other drawbacks of this index can be found elsewhere [41–43]. Our findings demonstrate that THI is in fact reliable as a unidimensional scale (with no subscales) in our large sample tinnitus sufferers, and its precision of measurement is the highest for subjects with moderate complaints.

Mokken analysis confirmed the unidimensionality of THI and allows us to treat it as a reliable cumulative scale. On the basis on several combined criteria, we propose that five items (THI2, THI8, THI13, THI19, THI24) should be removed in order to refine the scale. Three of these excess items belong to the original Functional subscale, while two belong to the Catastrophic subscale. Of the remaining 20 items, the majority cover the emotional aspect of tinnitus. This allows the whole scale to be more consistent, but it does narrow the range of tinnitus which THI measures. Kennedy and colleagues [60] noted that THI, compared to other tinnitus-related questionnaires, contains a disproportionately large number of items related to psychological/emotional aspects of tinnitus. The results of our study also suggest that tinnitus severity as measured by THI captures mainly the emotional aspects of tinnitus. This may be either a disadvantage or an advantage, depending on whether THI is used in a clinical or research setting and the underlying goal.

We must admit, that application IRT models to the THI posed some difficulties. Model fit statistic (M2) was significant for all tested models. It needs some comment [61], just like significant χ^2^ test values in previous analyses. First of all, CTT and IRT models represent an accept-support approach to model testing, where many “near perfect” models tend to be falsely “rejected”. Secondly, the χ^2^ statistic is generally susceptible to sample size therefore RMSEA, incremental fit indices and inspection of residuals and residuals correlations were developed and used to support model fit. Thirdly, the IRT models are predominantly psychometric not pure statistical/econometric models, therefore are focused on quality of data (given IRT model) rather than quality of model itself and model improving through its far-reaching respecification. Additionally, the problem of local independence should be also addressed. We used Yen’s *Q*_3_ statistic, however as it was shown by Christensen et al. [62] a singular critical value for *Q*_3_ is not fully appropriate and local dependence should be rather considered relative to the average observed residual correlation.

A great advantage and practical application of IRT is in-depth analysis of individual items, which may be used in selecting items during development or refinement of a questionnaire. Item location (level of difficulty) reflects where along the scale the item functions best. Items displaying a low level of item location (e.g. THI6 –*complaining a great deal about tinnitus*) are the ‘easiest’ items, indicating endorsement of mild tinnitus severity, while items with high item location (e.g. THI17 –*bad social relationship*) are the ‘hardest’ and they target a higher level of tinnitus severity. Informative items and discrimination were highest for THI21 (*depression*), THI14 (*irritation*), THI12 (*difficulty to enjoy life*), THI23 (*can no longer cope with tinnitus*); while the lowest were for THI11 (*having a terrible disease*) and THI7 (*trouble with sleep*). IRT parameters indicate which items should be selected to optimize measurement precision and achieve the desired goal of the tool. Items providing more information on lower-level traits are suitable for gauging mild tinnitus severity, while items targeting higher-level traits should be selected to optimize measurement of high tinnitus severity, e.g. in monitoring change over time following treatment. Item information function of THI displayed in Fig 7 clearly shows that THI in its present form is good in assessing individuals in the range Θ = –1 to 1, i.e. those with a moderate level of tinnitus severity.

Our findings have important clinical and research implications. The unidimensional factor structure of THI allows clinicians to use the tool without unnecessary additional calculations for subscales, thus saving time. Clinicians or researchers should rely only on the global score, because validity of the three subscales (Functional, Emotional, Catastrophic) is questionable, as they appear to provide little information beyond the general factor (overall tinnitus severity). We conclude that the quality of THI in its current form (25 items) is not satisfactory. Newman and colleagues proposed a short version of THI consisting of only 10 items [31], but they were selected on the basis of just three criteria: a high item–total correlation, representativeness of the three content domains, and face validity. We find such criteria insufficient and propose refining the THI instrument by removing just those items with some degree of misfit. We think that short form questionnaires are essential in busy clinical practice and with extensive research protocols, and we recommend taking into account both the CTT and IRT approaches in constructing a short form of THI.

The strength of our THI study is the large sample of tinnitus patients–the largest assembled so far. Patients came from all over Poland to our tinnitus clinic, so the sample can be considered representative of individuals seeking help for tinnitus. However, it is true that a more heterogeneous sample (e.g. in terms of geographic origin) would reduce the potential selection bias that our data might have.

We admit that not all aspects of IRT analysis have been exhausted in this study. Differential Item Functioning (DIF) analysis was omitted due to constraints on the length of this paper. Therefore, we still are unable to say how to interpret between-group comparisons shown with THI (e.g. difference in tinnitus severity between women and men) as true difference or measurement artifact. Further research is needed to establish measurement invariance in various demographic settings and cross-cultural comparisons.

To conclude, the growth of patient-centered care requires high-quality data from Patient Reported Outcome Measures. Application of IRT theory enables more precise assessment of the THI measurement properties, so that clinicians and researchers can have more confidence about their diagnoses and the results of trials based on THI.

We hope our findings might encourage researchers to use the IRT approach to explore the psychometric properties of other tinnitus-related questionnaires. Done well, we expect it will improve the quality of measures based on patients’ perception of their ailment.

## Supporting information

S1 Raw data (XLSX)

## References

[1] Food and Drug Administration. Guidance for Industry. FDA; 2009.

[2] Cappelleri J, Zou K, Bushmakin A, Alvir J, Alemayehu D, Symonds T. Patient-Reported Outcomes: Measurement, Implementation and Interpretation. Chapman and Hall/CRC; 2014.

[3] Chen J, Ou L, Hollis SJ. A systematic review of the impact of routine collection of patient reported outcome measures on patients, providers and health organisations in an oncologic setting. BMC Health Serv Res. 2013;13:211 10.1186/1472-6963-13-211 23758898 PMC3700832

[4] Mercieca-Bebber R, King MT, Calvert MJ, Stockler MR, Friedlander M. The importance of patient-reported outcomes in clinical trials and strategies for future optimization. Patient Relat Outcome Meas. 2018;9:353–67. 10.2147/PROM.S156279 30464666 PMC6219423

[5] Garratt AM, Bjaertnes ØA, Krogstad U, Gulbrandsen P. The OutPatient Experiences Questionnaire (OPEQ): data quality, reliability, and validity in patients attending 52 Norwegian hospitals. Qual Saf Health Care. 2005;14(6):433–7. 10.1136/qshc.2005.014423 16326790 PMC1744107

[6] Nelson EC, Eftimovska E, Lind C, Hager A, Wasson JH, Lindblad S. Patient reported outcome measures in practice. BMJ. 2015;350:g7818 10.1136/bmj.g7818 25670183

[7] Snyder CF, Jensen RE, Segal JB, Wu AW. Patient-reported outcomes (PROs): putting the patient perspective in patient-centered outcomes research. Med Care. 2013;51(8 Suppl 3):S73–79. 23774513 10.1097/MLR.0b013e31829b1d84 PMC3771694

[8] Aaronson N, Alonso J, Burnam A, Lohr KN, Patrick DL, Perrin E, et al Assessing health status and quality-of-life instruments: attributes and review criteria. Qual Life Res. 2002;11(3):193–205. 10.1023/a:1015291021312 12074258

[9] Terwee CB, Bot SDM, de Boer MR, van der Windt DAWM, Knol DL, Dekker J, et al Quality criteria were proposed for measurement properties of health status questionnaires. J Clin Epidemiol. 2007;60(1):34–42. 10.1016/j.jclinepi.2006.03.012 17161752

[10] Valderas JM, Ferrer M, Mendívil J, Garin O, Rajmil L, Herdman M, et al Development of EMPRO: a tool for the standardized assessment of patient-reported outcome measures. Value Health. 2008;11(4):700–8. 10.1111/j.1524-4733.2007.00309.x 18194398

[11] Mokkink LB, Terwee CB, Patrick DL, Alonso J, Stratford PW, Knol DL, et al The COSMIN checklist for assessing the methodological quality of studies on measurement properties of health status measurement instruments: an international Delphi study. Qual Life Res. 2010;19(4):539–49. 10.1007/s11136-010-9606-8 20169472 PMC2852520

[12] Møller AR. Introduction In: Møller AR, Langguth B, DeRidder D, Kleinjung T, editors. Textbook of Tinnitus. New York: Springer-Verlag; 2011 pp. 3–7.

[13] Tunkel DE, Bauer CA, Sun GH, Rosenfeld RM, Chandrasekhar SS, Cunningham ER, et al Clinical practice guideline: tinnitus. Otolaryngol Head Neck Surg. 2014;151(2 Suppl):S1–40. 25273878 10.1177/0194599814545325

[14] Jastreboff PJ. 25 years of tinnitus retraining therapy. HNO. 2015;63(4):307–11. 10.1007/s00106-014-2979-1 25862626

[15] Møller AR. Epidemiology of tinnitus in adults In: Møller AR, Langguth B, DeRidder D, Kleinjung T, editors. Textbook of Tinnitus. New York: Springer-Verlag; 2011 pp. 29–37.

[16] Savastano M. Characteristics of tinnitus in childhood. Eur J Pediatr. 2007;166(8):797–801. 10.1007/s00431-006-0320-z 17109163

[17] Skarzynski PH, Kochanek K, Skarzynski H, Senderski A, Szkielkowska A, Bartnik G, et al Hearing Screening Program in School-Age Children in Western Poland. Int Adv Otol. 2011; 7(2):194–200.

[18] Piotrowska A, Raj-Koziak D, Lorens A, Skarżyński H. Tinnitus reported by children aged 7 and 12 years. Int J Pediat Otorhinolaryngolog. 2015;79(8):1346–50. 10.1016/j.ijporl.2015.06.008 26104480

[19] Langguth B. A review of tinnitus symptoms beyond „ringing in the ears”: a call to action. Curr Med Res Opin. 2011;27(8):1635–43. 10.1185/03007995.2011.595781 21699365

[20] Zeman F, Koller M, Langguth B, Landgrebe M. Which tinnitus-related aspects are relevant for quality of life and depression: results from a large international multicentre sample. Health Qual Life Outcomes. 2014;12:7 10.1186/1477-7525-12-7 24422941 PMC3896823

[21] Langguth B, Goodey R, Azevedo A, Bjorne A, Cacace A, Crocetti A, et al Consensus for tinnitus patient assessment and treatment outcome measurement: Tinnitus Research Initiative meeting, Regensburg, July 2006. Prog Brain Res. 2007;166:525–36. 10.1016/S0079-6123(07)66050-6 17956816 PMC4283806

[22] Meikle MB, Stewart BJ, Griest SE, Henry JA. Tinnitus Outcomes Assessment. Trends Amplif. 2008;12(3):223–35. 10.1177/1084713808319943 18599500 PMC4134890

[23] Fackrell K, Hall D, Barry J, Hoare D. Tools for tinnitus measurement: development and validity of questionnaires to assess handicap and treatment effects IN: Signorelli F, Turjman F, editors. Tinnitus: causes, treatment and short and long-term health effects. New York: Nova Biomedical; 2014 pp. 13–60.

[24] Cima DRFF, Mazurek PDB, Haider H, Kikidis D, Lapira A, Noreña AJ, et al A multidisciplinary European guideline for tinnitus: diagnostics, assessment, and treatment. HNO. 2019;67:10–42. 10.1007/s00106-019-0633-7 30847513

[25] Skarżyński Piotr H., Rajchel Joanna J., Gos Elżbieta, Dziendziel Beata, Kutyba Justyna, Świerniak Weronika, et al A revised grading system for the Tinnitus Handicap inventory based on a large clinical population. Int J Audiol. 2020; 59(1):61–67. 10.1080/14992027.2019.1664778 31608728

[26] Newman CW, Jacobson GP, Spitzer JB. Development of the Tinnitus Handicap Inventory. Arch Otolaryngol Head Neck Surg. 1996;122(2):143–8. 10.1001/archotol.1996.01890140029007 8630207

[27] Salviati M, Macrì F, Terlizzi S, Melcore C, Provenzano A, Capparelli E, et al The Tinnitus Handicap Inventory as a screening test for psychiatric comorbidity in patients with tinnitus. Psychosomatics. 2013;54(3):248–56. 10.1016/j.psym.2012.05.007 23219227

[28] McCombe A, Baguley D, Coles R, McKenna L, McKinney C, Windle-Taylor P, et al Guidelines for the grading of tinnitus severity: the results of a working group commissioned by the British Association of Otolaryngologists, Head and Neck Surgeons, 1999. Clin Otolaryngol Allied Sci. 2001;26(5):388–93. 10.1046/j.1365-2273.2001.00490.x 11678946

[29] Gudex C, Skellgaard PH, West T, Sørensen J. Effectiveness of a tinnitus management programme: a 2-year follow-up study. BMC Ear Nose Throat Disord. 2009;9:6 10.1186/1472-6815-9-6 19558680 PMC2711043

[30] Zeman F, Koller M, Figueiredo R, Aazevedo A, Rates M, Coelho C, et al Tinnitus handicap inventory for evaluating treatment effects: which changes are clinically relevant? Otolaryngol Head Neck Surg. 2011;145(2):282–7. 10.1177/0194599811403882 21493265

[31] Newman CW, Sandridge SA, Bolek L. Development and psychometric adequacy of the screening version of the tinnitus handicap inventory. Otol Neurotol. 2008;29(3):276–81. 10.1097/MAO.0b013e31816569c4 18277308

[32] Zachariae R, Mirz F, Johansen LV, Andersen SE, Bjerring P, Pedersen CB. Reliability and validity of a Danish adaptation of the Tinnitus Handicap Inventory. Scand Audiol. 2000;29(1):37–43. 10.1080/010503900424589 10718675

[33] Baguley DM, Andersson G. Factor analysis of the Tinnitus Handicap Inventory. Am J Audiol. 2003;12(1):31–4. 10.1044/1059-0889(2003/007) 12894865

[34] Oron Y, Sergeeva NV, Kazlak M, Barbalat I, Spevak S, Lopatin AS, et al A Russian adaptation of the tinnitus handicap inventory. International Journal of Audiology. 2015;54(7):485–9. 10.3109/14992027.2014.996823 25620408

[35] Kleinstäuber M, Frank I, Weise C. A confirmatory factor analytic validation of the Tinnitus Handicap Inventory. J Psychosom Res. 2015;78(3):277–84. 10.1016/j.jpsychores.2014.12.001 25582803

[36] Meng Z, Zheng Y, Liu S, Wang K, Kong X, Tao Y, et al Reliability and validity of the chinese (mandarin) tinnitus handicap inventory. Clin Exp Otorhinolaryngol. 2012;5(1):10–6. 10.3342/ceo.2012.5.1.10 22468196 PMC3314799

[37] Bolduc D, Désilets F, Tardif M, Leroux T. Validation of a French (Québec) version of the Tinnitus Handicap Inventory. Int J Audiol. 2014;53(12):903–9. 10.3109/14992027.2014.935495 25140601

[38] Skarzynski PH, Raj-Koziak D, J Rajchel J, Pilka A, Wlodarczyk AW, Skarzynski H. Adaptation of the Tinnitus Handicap Inventory into Polish and its testing on a clinical population of tinnitus sufferers. Int J Audiol. 2017;56(10):711–5. 10.1080/14992027.2017.1319080 28537137

[39] Aqeel M, Ahmed A. Translation, Adaptation and Cross Language Validation of Tinnitus Handicap Inventory in Urdu. J Audiol Otol. 2017;22(1):13–9. 10.7874/jao.2017.00108 29325390 PMC5784365

[40] Cronbach LJ. Coefficient alpha and the internal structure of tests. Psychometrika. 1951;16(3):297–334.

[41] Schmitt N. Uses and abuses of coefficient alpha. Psychological Assessment. 1996;8(4):350–3.

[42] Sijtsma K. On the Use, the Misuse, and the Very Limited Usefulness of Cronbach’s Alpha. Psychometrika. 2009;74(1):107–20. 10.1007/s11336-008-9101-0 20037639 PMC2792363

[43] Dunn TJ, Baguley T, Brunsden V. From alpha to omega: a practical solution to the pervasive problem of internal consistency estimation. Br J Psychol. 2014;105(3):399–412. 10.1111/bjop.12046 24844115

[44] Hooper D, Coughlan J, Mullen M. Structural Equation Modelling: Guidelines for Determining Model Fit. Articles [Internet]. 1 styczeń 2008; Available from://arrow.dit.ie/buschmanart/2

[45] Sagan A. Analiza rzetelności skal w wielopoziomowych modelach pomiaru (Reliability Analysis in Multilevel Measurement Models) In: Prace Naukowe Uniwersytetu Ekonomicznego we Wrocławiu (Research Papers of Wrocław University of Economics). Wrocław: Uniwersytet Ekonomiczny we Wrocławiu; 2014 pp. 49–59.

[46] Rodriguez A, Reise SP, Haviland MG. Evaluating bifactor models: Calculating and interpreting statistical indices. Psychol Methods. 2016;21(2):137–50. 10.1037/met0000045 26523435

[47] Rodríguez AC, Reise SP, Haviland MG. Applying Bifactor Statistical Indices in the Evaluation of Psychological Measures. J Pers Assess. 2016;98(3):223–37. 10.1080/00223891.2015.1089249 26514921

[48] Reise SP, Bonifay WE, Haviland MG. Scoring and modeling psychological measures in the presence of multidimensionality. J Pers Assess. 2013;95(2):129–40. 10.1080/00223891.2012.725437 23030794

[49] Fornell C, Larcker DF. Evaluating Structural Equation Models with Unobservable Variables and Measurement Error. J Market Res. 1981;18(1):39–50.

[50] Bentler P. Covariance Structure Models for Maximal Reliability of Unit-Weighted Composites In: Lee S, editor. Handbook of Latent Variable and Related Models. New York: Elsevier; 2007 pp. 1–19.

[51] Hancock G, Mueller R. Rethinking Construct Reliability within Latent Variable Systems In: Cudeck R, du Toit S, Sorbom D, editors. Structural Equation Modeling: Present und Future—A Festschrift in Honor of Karl Joreskog. Lincolnwood, IL: Scientific Software International; 2001 pp. 195–216.

[52] Reise SP. The rediscovery of bifactor measurement models. Multivar Behav Res. 2012;47(5):667–96. 10.1080/00273171.2012.715555 PMC3773879 24049214

[53] Stucky BD, Thissen D, Edelen MO. Using Logistic Approximations of Marginal Trace Lines to Develop Short Assessments. Appl Psychol Meas. 2013; 37(1):41–57.

[54] Muthén B, Kaplan D, Hollis M. On structural equation modeling with data that are not missing completely at random. Psychometrika. 1987;52(3):431–62.

[55] Mokken RJ. A Theory and Procedure of Scale Analysis, With Applications in Political Research [Internet]. Reprint 2011. Berlin, Boston: De Gruyter Mouton; 2011 Available on: https://www.degruyter.com/view/product/46584

[56] Muraki E. A Generalized Partial Credit Model: Application of an Em Algorithm. ETS Research Report Series. 1992;(1):i–30.

[57] Samejima F. Estimation of Latent Ability Using a Response Pattern of Graded Scores1. ETS Research Bulletin Series. 1968;1968(1):i–169.

[58] Maydeu-Olivares A, Joe H. Limited- and Full-Information Estimation and Goodness-of-Fit Testing in 2n Contingency Tables. Journal of the American Statistical Association. 2005;100(471):1009–20.

[59] Yen WM. Effects of local item dependence on the fit and equating performance of the three-parameter logistic model. Appl Psychol Meas. 1984;8(2):125–45.

[60] Kennedy V, Wilson C, Stephens D. Quality of life and tinnitus. Audiol Med 2009;2:29–40.

[61] Barrett P. Structural equation modelling: Adjudging model fit. Pers Individ Dif. 2007;42(5):815–24.

[62] Christensen KB, Makransky G, Horton M. Critical Values for Yen’s Q3: Identification of Local Dependence in the Rasch Model Using Residual Correlations. Appl Psychol Meas. 2017;41(3):178–94. 10.1177/0146621616677520 29881087 PMC5978551

